# Supplementary material for: Association between oral microbiome and seven types of cancers in East Asian population: a two-sample Mendelian randomization analysis
Source: Front Mol Biosci. 2023 Nov 21;10:1327893. doi: 10.3389/fmolb.2023.1327893 (PMC10702768; doi:10.3389/fmolb.2023.1327893)
Supplement: Supplementary file 1 [file DataSheet1.ZIP › Supplementary Materials/MR plots for tongue/tongue═╝/Ovarian cancer/pheno.342_to_ovarian cancer_leave_one_out.pdf]

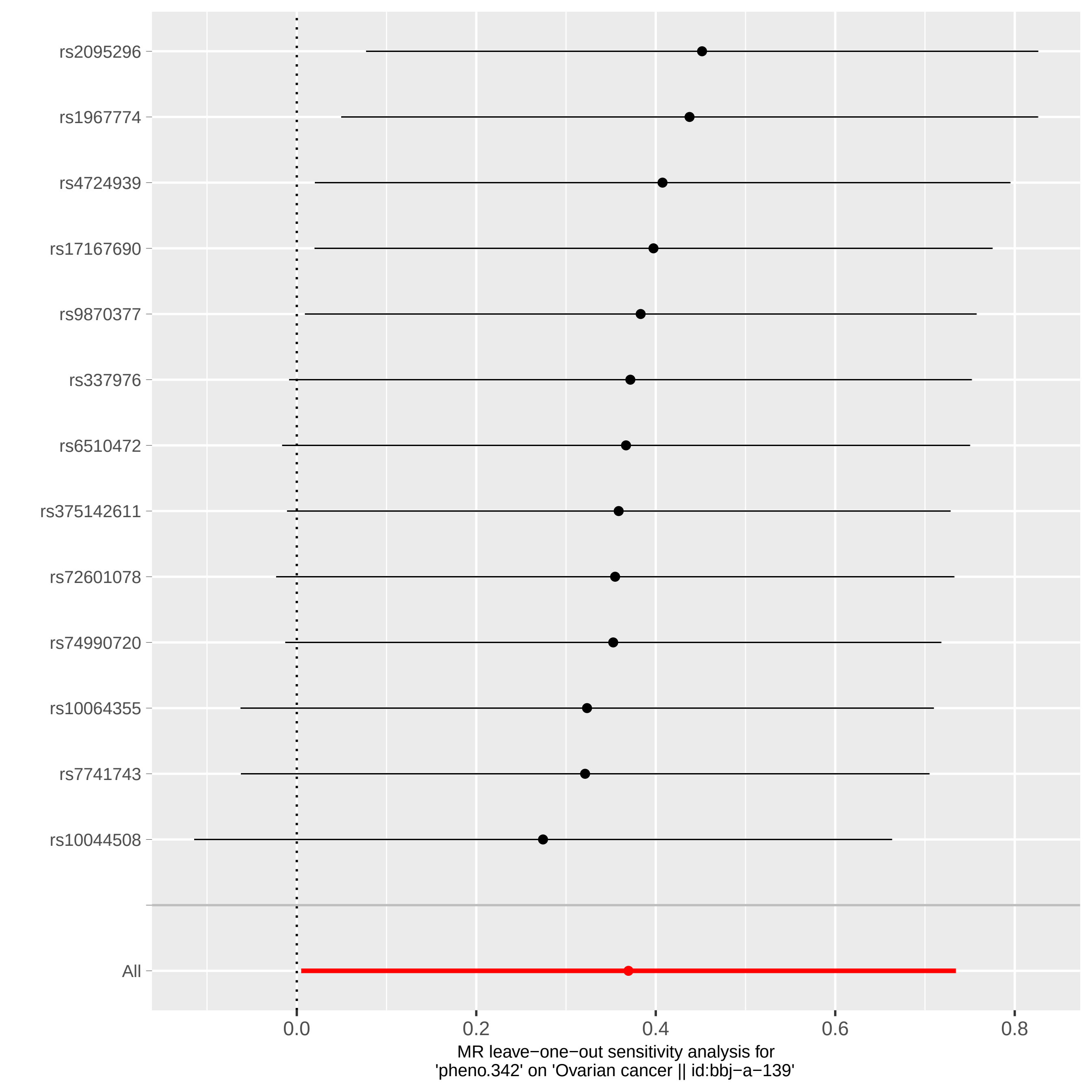
rs2095296
rs1967774
rs4724939
rs17167690
rs9870377
rs337976
rs6510472
rs375142611
rs72601078
rs74990720
rs10064355
rs7741743
rs10044508
All
0.0
0.2
0.4
0.6
0.8
MR leave−one−out sensitivity analysis for
'pheno.342' on 'Ovarian cancer || id:bbj−a−139'
